# Impact of Gluten-Free and Casein-Free Diet on Behavioural Outcomes and Quality of Life of Autistic Children and Adolescents: A Scoping Review

**DOI:** 10.3390/children11070862

**Published:** 2024-07-16

**Authors:** Kristina Zafirovski, Mirjana Trpevska Aleksoska, Joe Thomas, Fahad Hanna

**Affiliations:** 1 Program of Public Health, Department of Health and Education, Torrens University Australia, Melbourne, VIC 3000, Australia; kristina.zafirovski@torrens.edu.au (K.Z.); mirjana.aleksoska1@health.torrens.edu.au (M.T.A.); 2 Department of Public Health, Institute of Health and Management, Level 2/187 Boundary Rd, North Melbourne, VIC 3051, Australia; joe.t@ihm.edu.au

**Keywords:** autism spectrum disorders (ASD), autistic symptoms, gluten-free diet, casein-free diet, quality of life, children and adolescents

## Abstract

Background: Gluten- and casein-containing foods could aggravate the symptoms of children and adolescents with autism spectrum disorder (ASD), and subsequently impact their quality of life. However, there is a mixed opinion among researchers concerning the impact of alternative diet on reducing ASD symptoms. Objective: This scoping review aimed at examining the impact of the “gluten-free, casein-free” (GFCF) diet on health outcomes and the quality of life among autistic children and adolescents. Methods: A scoping review of the literature was performed following the Joanna Briggs Institute (JBI) guidelines. Four databases, including EbscoHost, Medline, CINAHL, and ProQuest, were used to obtain subject-specific studies relevant to the research question and published between July 2013 and March 2024. A comprehensive search using keywords such as “autism spectrum disorder”, “gluten-free diet”, and “casein-free diet” was conducted to obtain articles related to the research focus area. Only full-text, peer-reviewed, written in English articles were selected. Data extraction and data analysis were performed according to the Preferred Reporting Items for Systematic Reviews and Meta-Analyses-extension to Scoping Review (PRISMA-ScR) protocol. Results: From the initial 586 studies, a total of 27 articles were included in the final analysis of the review. The thematic analysis included “GFCF diet and improvement of the core autistic symptoms”, “the gut–brain link”, “dietary interventions and autism”, “possible side effects due to the GCFC diet”, and “inconclusive studies and mixed opinions”. A majority of the studies showed a positive effect of the GFCF diet on a variety of autistic symptoms, including positive changes in cognitive skills, behaviour, and gastrointestinal symptoms, while some showed conflicting evidence. Conclusions: The currently available evidence on the impact of the “GFCF” diet on the quality of life of autistic children and adolescents may warrant potentially effective interventions for alleviating symptoms of autism spectrum disorders. However, this scoping review highlights the need for more research to provide more reliable evidence on the health outcomes and quality of life of ASD sufferers to guide practice.

## 1. Introduction

According to the World Health Organisation (WHO), autism spectrum disorder (ASD), or autism, represents a diverse group of conditions associated with difficulties in the development of the brain [1]. ASD is characterised by difficulty in communication, sensory anomalies, stereotypic and repetitive behaviours, and varying degrees of intellectual disabilities [2]. The development of ASD is multifactorial and could be caused by genetic or environmental factors alone or as a combination of both [3]. Currently, no medical tests are available to diagnose the disorder [4] and can be established by experienced health professional assessment of the child’s activity by the age of two [5]. This diagnosis of ASD is based on typical characteristics of autism, including repetitive behaviours and impaired social communication and interaction [4]. Despite the core symptoms, autism is often accompanied by comorbidities including psychiatric/behavioural complaints and gastrointestinal (GI) disorders [6]. Given the variation in children’s manifestations of ASD, degrees of comorbid conditions, as well as differences in level of functioning for children, the treatment recommendations are of vital importance for practitioners [7].

Epidemiological data from WHO in 2023 showed an increasing trend of autism globally, where 1 in 100 children suffers from autism, with the occurrence being four times higher in boys than girls [1]. It is evident that ASD symptoms affect each person differently and require a different treatment approach [2]. Treatments for autism usually involve two interventions: comprehensive treatment models with structured programs, and focused intervention practices designed to treat behavioural symptoms [8]. Nevertheless, despite advancements in treatments and early diagnosis of ASD, the core symptoms of autism cannot be reversed [9]. Consequently, families are seeking alternative treatments, such as restrictive diets, especially the gluten-free and casein-free (GFCF) diet, which displays promising effects in improving the quality of life among ASD children and adolescents by improving various autistic symptoms, including their communication, cognition, and stereotypical behaviour [10]. The GFCF diet involves eliminating products containing gluten (a protein found in wheat) and casein (a protein found in dairy products) [11]. There are several theories supporting the GFCF diet mechanisms in improving the symptoms of autism, among which the opioid excess hypothesis is the most prevalent [10]. The focus of this diet is the theory of the “leaky gut”, where the increased intestinal permeability allows gluten and casein peptides to leak from the gut, resulting in opioid activity causing ASD behaviours [12]. Additionally, children with autism spectrum disorders are more prone to gastrointestinal (GI) disorders than the general population, which can significantly impact their health, learning, and development [13]. Since gastrointestinal disorders may affect the incidence and severity of other symptoms in children with ASD, adequate nutrition is essential and can improve their life comfort and overall health [14]. Various studies suggested that the gluten-free diet may be effective in controlling gastrointestinal issues in children with ASD and improving their behaviour [15]. Contemplating the positive impact of the GFCF diet on improving the behaviour of autistic children, this GFCF diet could be considered as beneficial to enhance behavioural symptoms in these children [16]. 

Several knowledge gaps were discovered in the reviewed literature. For instance, it was evident that various researchers expressed opposite opinions about the effectiveness and safety of the GFCF diets [17]. Furthermore, the related literature lacks empirical evidence to support both theories. While many authors have demonstrated the benefits of administering the GFCF diet to reduce autism symptoms, others believe that following the GFCF could pose a significant risk to the wellbeing of autistic children. Risks associated with following the GFCF diet among autistic children, who already struggle with selective eating habits, could potentially cause nutrition deficit, weight loss, and sleeping disturbance [17,18]. Consequently, this scoping review aimed to critically analyse the impact of gluten-free/casein-free diet (GFCF) on the quality of life of autistic children and adolescents.

## 2. Methodology

A comprehensive scoping review utilising the “Joanna Briggs Institute (JBI) methodology for scoping review” was performed, which is a search framework first suggested by Arksey and O’Malley in 2005 [19,20]. The scoping review consists of the following steps: developing the research question, identifying the relevant literature, selecting the relevant literature that meets the inclusion criteria, extracting results, and presenting these results [19]. A scoping review approach was chosen for this study, given the aim and objectives of the research. A scoping review design helps adopt a broader research strategy while also ensuring the reproducibility, transparency, and reliability of existing knowledge in the field. The review followed the methodology outlined by the Joanna Briggs Institute and adhered to the reporting guidelines provided by the Preferred Reporting Items for Systematic Reviews and Meta-Analyses-extension for Scoping Reviews (PRISMA-ScR) statement [21]. The search strategy employed an iterative process, and was guided by the primary question: How does the adoption of the GFCF diet affect the quality of life of autistic children and adolescents?”

Database search:

A literature search was conducted in MEDLINE, EBSCOHost, CINAHL, and ProQuest to identify papers published between July 2013 and March 2024 using a combination of keywords and MESH terms for autism spectrum disorders AND gluten-free diet AND casein-free diet. Multiple databases were chosen for this study to improve results and reduce the risk of overlooking any eligible studies that could be used during our final appraisal [22].

Titles and abstracts of studies were first screened against the above inclusion criteria to determine which articles would undergo full-text review. Then, the full text of the resulting papers was reviewed for inclusion. Furthermore, the reference list of all included articles was searched for additional articles. Exclusion criteria included studies that were released before July 2013, and performed on participants who did not match the age group of children and adolescents. We included peer-reviewed studies that provided original data (e.g., randomised controlled trials and observational studies) as well as systematic reviews. After data extraction was completed, Braun and Clarke’s approach to thematic analysis was used to evaluate the data [23]. The approach consisted of six steps: 1. Being familiar with the data; 2. Producing initial codes for the data; 3. Searching for potential themes; 4. Reviewing themes; 5. Defining and naming themes; and 6. Reporting and analysing themes [23]. Phase 6 was completed using PRISMA-ScR guidelines [21].

### 2.1. Choice of Design

See Figure 1.

A scoping review was preferred as it helps to adopt a broader search strategy while also allowing reproducibility, transparency, and reliability of the existing knowledge in the field [20]. Hence, it is a valuable method to assess the effects of a GFCF diet on children with autism spectrum disorders. Moreover, scoping reviews can help map the fundamental concepts of a research area, define working definitions, and establish the limits of a topic [24]. Furthermore, a scoping review of other scoping reviews discovered that the primary bases for performing one are to understand the scope of the existing literature, provide a summary of the available evidence, and offer direction for future studies [20].

### 2.2. Data Sources

A systematic search was performed on four databases, including EbscoHost, Medline, CINAHL, and ProQuest, from July 2013 to March 2024. Furthermore, the review included only studies that examined the use and importance of a GFCF diet for children and adolescents with ASD. A comprehensive search using keywords such as “autism spectrum disorder, gluten-free diet, and casein-free diet” was conducted to obtain articles related to the research focus area. The review process only considered full-text, peer-reviewed articles, written in English that analyse the effects of the GFCF diet on ASD symptoms and behaviours in children and adolescents with ASD. 

### 2.3. Study Selection

A standardised process of screening and selecting studies to create a comprehensive and inclusive final list was performed. After the initial identification of studies, duplicates were eliminated and articles that did not meet our criteria for inclusion and exclusion were excluded. We included articles with the following criteria: (1) Studies that investigated or evaluated data on a gluten-free diet and/or casein-free diet for autistic children and adolescents; (2) Children/adolescents with or without comorbidities. Additionally, we included both qualitative (case–control, cross-sectional, narrative reviews, and systematic reviews) and quantitative studies (meta-analysis, randomised control trials) in this review. We excluded articles published before July 2013. Afterwards, a thorough full-text screening was conducted to determine the final list of included studies. The PRISMA-ScR checklist was used to carry out the screening process and to propose studies pertinent to this scoping review’s inclusion/exclusion criteria [25]. 

The protocol for this scoping review was not published or registered prior to conducting the scoping review.

## 3. Results

**Figure 1:**
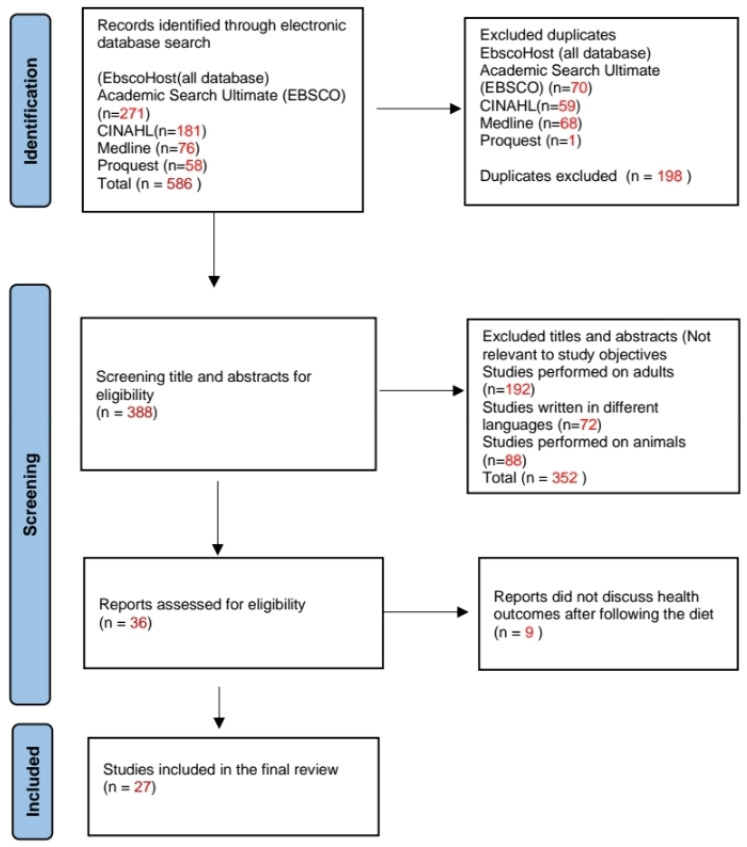
PRISMA flow chart of the selection process.

The literature search resulted in 586 full-text articles (Figure 1). Following the removal of 199 duplicates, 388 articles were further screened (titles and abstracts) for eligibility, which resulted in the removal of an additional 352 articles due to not being relevant to the study objectives. The remaining 36 reports were then screened for full text, resulting in the exclusion of an additional 9 articles due to the poor quality of the articles. A total of 27 studies were included for analysis in this scoping review. Figure 1 provides a chart of the screening process.

### Data Charting

Following the JBI protocol of 2022, the data extraction or charting process delivers crucial insights to the reader, including a comprehensive overview of the outcomes that address the scoping review inquiries [20]. Following the finalisation of the screening process, the data extraction process was performed. A data charting table was completed, and the data extraction was performed by two reviewers (K.Z. and M.T.). Each study was evaluated based on its eligibility criteria for inclusion or exclusion in this review and based on the agreement of both reviewers. Whenever an agreement was not reached, F.H.’s view was sought for the final decision. 

Looking at the data extraction table (Table 1), the main information included records for each source. This included the reference and any results or findings that were relevant to the review question [20]. During the review stage, the charting table was revised accordingly. The selected studies for our scoping review were then organised based on the year of study, parameters used, and population type (children and adolescents). Following this, the reviewers provided a detailed description of each study that was included, outlining the study reference (author, year, country), the sample population (if applicable), the aim of the study, study designs, the methodology used, parameters, key findings, and summary of findings. Both reviewers reached a complete consensus on all the extracted data. The charting table was consistently revised to facilitate ongoing data enhancement.

## 4. Thematic Analysis

There are several advantages to performing secondary data analysis. One of these is the economic benefit, as the data have already been collected and stored electronically. This allows researchers to focus their time and resources on data analysis rather than collecting it [51]. Moreover, researchers can utilise the available data to test hypotheses and achieve their research objectives [52]. Thematic analysis is a widely used and valuable approach that systematically detects and categorises patterns or themes within a data collection [23]. In 2020, Crosley explained that qualitative data coding involves creating and assigning codes to organise extracted data [53]. These codes, also known as keywords, are used to identify themes and patterns for thematic analysis, which can be performed simultaneously with coding and analysis. The process of thematic analysis involves identifying patterns of meaning, also known as themes, within our resources. These themes help us answer the study’s main research questions by coding our data and developing relevant themes subsequently [23]. Several codes and themes based on the extracted data were observed during the data analysis process. One noticeable finding was the impact of the GFCF diet on various aspects of the quality of life in individuals with autism. In addition, the following themes were identified. 

### 4.1. GFCF Diet and Improvement of the Core Autistic Symptoms

#### 4.1.1. Behaviour Improvements in Children with ASD Who Presented Gastrointestinal Symptoms

Three studies showed behaviour improvement among ASD children presenting with gastrointestinal issues [27,30,46]. An experimental survey conducted on 30 ASD children showed that only autistic children who present both with very high urinary peptide and gastrointestinal problems respond positively to a gluten-free, casein-free diet [46]. Additionally, the GFCF diet was effective in improving the behaviours of children with ASD who presented gastrointestinal symptoms, specifically constipation and diarrhoea, compared to those without gastrointestinal disease [27,30]. Likewise, implementing the GFCF diet for 3–6 months among children with ASD presenting with gastrointestinal symptoms showed improvement in cognitive, behavioural, social, communication, motor, and gastrointestinal symptoms [30].

#### 4.1.2. Improvements in Sleeping Patterns

The impact of sleeping problems on the behaviour of autistic children has been extensively researched. Four studies showed that removing gluten- and casein-containing foods from the diets of individuals with autism improved sleeping issues [28,31,41,42]. Additionally, significant improvements have been noted in behaviour, vocal and nonvocal communication, attention and concentration, episodes of aggressiveness, affection, motor skills, sleeping patterns, displaying of routines and rituals, anxiety, empathy, and responses to learning [31].

#### 4.1.3. Communication Improvements

Communication improvements were observed in six studies [31,32,37,39,43,50]. A randomised, controlled, single-masked 12-month study suggested that a comprehensive nutritional and dietary intervention showed statistically significant benefits in communication subscores in the GFCF diet group compared to the control group [43]. Moreover, in a systematic review conducted in Denmark, six randomised controlled trials involving 214 participants were analysed to determine the effectiveness of a GFCF diet on the symptoms of ASD in children [32]. One trial resulted in significant improvements in the communication subdomain of the Autism Diagnostic Observation Schedule (ADOS) scores in the GFCF diet group compared to the control group [32]. Additionally, a case–control study found that implementing a GFCF diet resulted in a reduction in the severity of symptoms related to ASD and improved scores in the areas of speech, language, and communication [37]. Furthermore, a recent systematic review discovered that interventions for children and adolescents (aged 0–19) resulted in moderate improvements in communication, stereotyped movements, aggressiveness, and symptoms of attention-deficit/hyperactivity disorder (ADHD) [39]. Lastly, a review of systematic reviews on children with ASD found that the GFCF diet was highly beneficial in improving communication and interaction abilities [47]. Two small randomised controlled trials (RCTs) involving 35 participants showed significant treatment effects in favour of the dietary intervention for overall autistic traits and the ability to communicate and interact [47]. Similarly, a systematic review on children with ASD found that the GFCF diet was highly beneficial in improving communication and interaction abilities [50]. 

#### 4.1.4. Stereotypic Activity Improvement

Five studies showed improved autistic stereotypical activity [10,35,39,43,50]. A 12-month randomised, controlled, and single-blind study examined the effect of GFCF on stereotypic behaviour [43]. The effects of the GFCF diet on ASD symptoms were observed, and the results showed significant improvement in nutritional statuses, nonverbal IQ, and autism symptoms such as social isolation, eye contact, mutism, learning skills, hyperactivity, stereotypic activity, and panic attacks [43]. Moreover, a systematic review showed improved communication and stereotypic movements and behaviour among ASD children and adolescents [39]. Recent systematic review and meta-analysis showed improved stereotypical autistic behaviour [10]. Additionally, recent studies showed improvement in stereotypical movements [35,50].

#### 4.1.5. Cognitive Improvements

Six studies identified positive changes in autistic behaviour [11,30,31,48,49,50]. Three studies showed improvement in cognitive, behavioural, and communication symptoms among children with ASD following the implementation of the GFCF diet [11,30,31]. In addition, concentration as a cognitive skill was improved (among 30% of the participants) in one experimental survey and two literature reviews [11,31]. A recent meta-analysis showed that there was a relationship between adherence to the GFCF diet and the behaviour of autistic children [48]. Additionally, a case–control study showed improvement in autistic behaviour [49]. Additionally, a systematic review and meta-analysis showed that the GFCF diet can reduce stereotypical behaviours and improve the cognition of children with ASD [50].

#### 4.1.6. Improvement in Attention Deficit Hyperactive Disorders (ADHD) Symptoms 

Six studies showed improvement in ADHD symptoms among autistic children and adolescents [35,39,42,43,45,50]. Additionally, it was found that the GFCF diet improved overall wellbeing and improved behaviour, cognitive function, learning abilities, developmental outcomes, and reduced hyperactive and anxious behaviour in autistic children [42]. A well-designed case–control study assessed the hyperactivity behaviours of 72 autistic children. It documented a positive effect primarily in reduced symptoms of hyperactivity/impulsivity and inattention, symptoms that are very common in children with ASD and can impact a child’s learning capacity [45]. Chronological age was the strongest predictor of response, where participants aged between 7 and 9 seemed to derive the most benefit from a dietary intervention. Moreover, a randomised, controlled, single-blind 12-month study reported that the parents (who were not blinded) announced benefits in social interaction, daily living skills, inattention, and hyperactivity [43]. Furthermore, a recent systematic review found moderate improvements in the symptoms of ADHD for individuals aged 0–19 [39]. Another systematic review showed that the GFCF diet is beneficial in improving ADHD symptoms and aggressiveness among autistic children [50]. Likewise, two additional studies demonstrated slight enhancements in hyperactivity [35,42]. 

### 4.2. The Gut–Brain Link

#### 4.2.1. GFCF Diet and Coeliac Disease (CD)

Three studies examined the correlation between coeliac disease, GFCF diet, and autism-related behaviours and symptoms of the participant [26,33,34]. A case–control analysis involving 140 paediatric patients investigated the potential correlation between autism and celiac disease by evaluating immune reactivity to gluten [33]. The findings revealed that dietary intake is linked to various behavioural and psychometric changes in some individuals with autism that affect core areas of communication and social interaction. Moreover, the GFCF diet benefits children with ASD who were diagnosed with celiac disease or food allergies because the diet helps to relieve gastrointestinal discomfort [34]. Additionally, it was concluded that the GFCF diet prevents immune response to gluten (antibodies against gluten in ASD are different from celiac disease) and casein, decreases urinary peptides, and improves autistic-related behaviour [26].

#### 4.2.2. The Opioid Theory

The function of the gut–brain axis was examined, and it was found that abnormalities in carbohydrate digestion and absorption could explain some of the gastrointestinal problems observed in a subset of ASD children between 6 and 17 years old [31]. Two studies supported the opioid theory and found a decrease in peptide levels and an improvement in autism symptoms after the GFCF diet implementation [26,41]. Furthermore, an experimental study was conducted on 30 children with autism spectrum disorder aged between 6 and 12 years old and found that the diet decreases the urinary peptide level for 40% of children [46].

### 4.3. Dietary Interventions and Autism

It was found that the GFCF diet improved overall wellbeing, as well as showed improvement in behaviour, cognitive function, learning abilities, developmental outcomes, and reduced hyperactive and anxious behaviour [32]. A small size RCT involving 35 participants showed significant treatment effects in favour of dietary intervention such as the GFCF diet for overall autistic traits [47]. Moreover, a recent systematic review showed that the GFCF diet is beneficial in improving communication, stereotypical movement, aggressiveness, hyperactivity, ADHD, and gastrointestinal issues among autistic children and adolescents [50]. An RCT using treatment involving special vitamin/mineral supplements, essential fatty acids, Epsom salt baths, carnitine, digestive enzymes, and a healthy gluten-free, casein-free, soy-free (HGCSF) diet showed significant improvement in nonverbal intellectual ability in the treatment group compared to the control group [43]. A recent meta-analysis also showed improvement in maladaptive behaviour among autistic children adhering to GFCF diet [48]. 

#### Ketogenic Diet, Modified Atkins Diet (MAD), and GFCF Diet

A case–control study involving 45 children (aged 3–8) showed that the ketogenic diet, modified Atkins diet (MAD), and GFCF diet could be implemented safely and may improve autistic symptoms [37]. This case–control study showed that the GFCF group showed improvement in speech and behaviour. 

### 4.4. Possible Side Effects Due to the GFCF Diet

A slight initial worsening in behaviour was noticed after the introduction of the GFCF diet among autistic children and adolescents, which was advised to be comparable with the withdrawal behaviours manifested by the removal of opioids [31]. A recent systematic review identified risks that could be associated with following the GFCF diet, such as lower anthropometric measures than children with ASD on a regular diet [38]. Additionally, the Healthy Eating Index (HEI) and nutrient intake of foods would differ between these groups, and children with ASD on a GFCF diet would not meet the daily recommended intake when compared to children with ASD on a regular diet [38]. A systematic review and meta-analysis reported side effects associated with the GFCF diet such as gastrointestinal discomfort, weight loss, waking up at night, and decreased appetite [40]. Furthermore, it was reported that long-term administration of the GFCF diet could cause micronutrient deficiencies and social isolation among ASD children [11].

### 4.5. Inconclusive Studies and Mixed Opinions

Despite the lack of empirical validation, the GFCF diet remains a widely used method to diminish ASD-related symptoms among individuals with ASD [30]. Trials involving the GFCF diet to improve ASD symptoms show contradictory but promising results [31]. However, the many possible mechanisms are not completely understood, with many contradicting results from an extensive number of studies making it even harder for parents and children to find sufficient help in dealing with ASD [31]. Six studies have found inconclusive evidence of the effect of a GFCF diet on behaviour [34,35,36,44,45,50]. Additionally, controversial effects of the GFCF diet were reported after long-term administration [11]. Similarly, the research data in this field of study are limited and not yet indicative of any population-wide effect to be had from such dietary changes [28,50].

## 5. Discussion

Our scoping review of global studies highlighted a positive impact of the GFCF diet on the quality of life of children and adolescents with ASD, with several studies having reported positive effects. In particular, evidence from this review suggests that individuals with ASD who are exposed to the GFCF diet may experience improvements in their behaviour, cognitive function, learning abilities, and developmental outcomes, as well as reduced hyperactivity. On the other hand, some researchers expressed mixed opinions and lack of scientific evidence pertinent to the effectiveness of the GFCF diet.

Improvement in communication skills among autistic children adhering to the GFCF diet was evident in several studies [31,32,37,39,43,50]. This finding is further supported by earlier studies outside the timeframe of this review that reported a reduction in autistic traits and increased communicative skills in children with ASD following the implementation of the GFCF diet [54]. Moreover, improvement in ADHD-associated symptoms among autistic children and adolescents after implementing the GFCF diet was also reported in various studies [35,39,42,43,45,50]. These findings are consistent with the results from a more recent meta-analysis that found that consuming foods containing gluten and casein may contribute to hyperactivity in autistic children [16]. The above meta-analysis went on to show a significant positive effect on the behaviour of autistic children, including anxiety, ADHD, aggressiveness, sleep issues, attention, and other autism-related symptoms [16].

This review found that a GFCF diet could reduce stereotypical behaviours and improve the cognition of children with ASD. Similarly, an Indonesian meta-analysis demonstrated that the GFCF diet reduced the risk of maladaptive behaviour in children with autism [48]. This consistency in the findings was also observed in the findings of an over 20-year-old single-blinded, controlled study that evaluated the effect of a GFCF diet on children with autism [54]. The trial was carried out for one year and showed a significant reduction in autistic behaviour for participants in the intervention group (GFCF diet) when compared to the control group. The above trial showed improved cognition, significant increase in willingness and abilities to communicate, and decreased autistic traits [54]. 

Implementing the GFCF diet among autistic children has also been shown to contribute to improved sleeping habits. An earlier study in India (2008) evaluated the impact of casein- and gluten-free diets among selected autistic children and showed improvement in sleeping patterns in these children [55]. Similarly, a recent study showed that implementing a gluten-free diet for six months significantly improved sleeping disturbances regardless of initial age, sex, and symptom status of the participants [56].

Interest in dietary therapies for diminishing ASD-related symptoms, such as the GFCF diet and ketogenic diet, is growing annually [57]. The findings of our studies showed that the ketogenic diet, modified Atkins diet (MAD), and GFCF diet could be implemented safely to improve autistic symptoms. These findings are consistent with the findings of a recent meta-analysis that suggested that gluten-free diets were beneficial to improving social behaviours, while the ketogenic diet (KD) suggested a significant effect on core autistic symptoms [57]. Furthermore, previous studies showed that the GFCF diet is the most effective dietary treatment for diminishing ASD-related behaviours, while the ketogenic diet improved behaviour and hyperactivity among autistic individuals [58]. 

While the benefit of the GFCF diet has been widely acknowledged and recommended as a practical guide to practitioners and families [59], some side effects associated with the implementation of the GFCF diet among autistic children were reported in our review. One systematic review reported risks such as lower anthropometric measures among autistic children following the GFCF diet when compared to autistic children on a regular diet [38]. Additionally, the above systematic review showed that children who adhered to the GFCF diet did not meet the recommended daily intake of nutrients when compared to children on a regular diet [38]. Similarly, a recent systematic review and meta-analysis identified side effects of the GFCF diet including gastrointestinal discomfort, weight loss, sleeping disturbance, and loss of appetite [40].

Despite a larger volume of studies supporting GFCF diet compared to those questioning it, the effects of the GFCF diet among ASD children and adolescents remain debatable. Therefore, it is paramount to rigorously evaluate both the efficacy and safety of a GFCF diet in children and adolescents with ASD prior to systematically implementing the diet. Further and more rigorous studies are still required to confirm our findings and to confidently make decisions on the recommendation, or otherwise, of this diet/intervention in practice. Healthcare providers and policymakers should consider this and engage in discussions tailored to each patient’s specific needs and characteristics.

## 6. Limitations

Our study has some limitations to consider. As a scoping review, the retrieved data were derived from various study designs and methods, some of which may have lacked rigor. Some of the included studies are characterised by a lack of sufficient methodological quality due to the low participant rates, low compliance rates, challenges in blinding the participants to which diet they were on, and inadequate autistic diagnosis. Despite our attempt to cover all literature within the specified review time, some studies may have been accidentally missed and may have impacted the overall findings and the summary of evidence here. Furthermore, only English-language publications and available full-text articles were considered due to language comprehension and access limitations. Another substantial limitation is the timeframe limitation to perform the study.

## 7. Conclusions

Based on the researched data, it was evident that the GFCF diet was one of the most accepted and applied treatments for reducing symptoms associated with ASD. Although many studies supported the effectiveness of the GFCF diet, some expressed opposite opinions. The review discussed the quality of life concerning several factors, including enhancement in behavioural problems, sleeping patterns, communication, and stereotypical activities. Additionally, it explored the advantages of the GFCF diet in alleviating gastrointestinal issues in children with ASD. Despite this, the available evidence is limited and does not provide consistent and robust findings to support the effectiveness of a GFCF diet in children diagnosed with ASD. Thus, clinicians must exercise prudence and consider various factors when recommending the GFCF diet. Additional large-scale, high-quality clinical trials of sufficient duration are necessary to further clarify the potential effects of the GFCF diet on the performance and functional outcomes of children and adolescents with ASD.

## Figures and Tables

**Table 1:** Characteristics of included studies.

Author, Year, Country	Study Title/Objectives	Sample Size	Aim	Methodology	Parameters	Key Findings	Summary of Findings
Liang et al., 2023 [26]. USA	Food, gut barrier dysfunction, and related diseases: A new target for future individualised disease prevention and management.	N/A	Describe the relationship between dietary factors, intestinal permeability dysfunction, and related diseases including ASD.	Literature review	Gut barrier dysfunction; behaviour.	GFCF diet prevents immune response to gluten and casein and improves behaviour.	There is strong evidence that dietary changes might offer therapeutic strategies to address gut barrier dysfunction.
Esposito et al., 2023 [27]. Italy	Food Selectivity in Children with Autism: Guidelines for Assessment and Clinical Interventions.	N/A	Provide evidence-based sensorial and behavioural strategies in addressing food selectivity in children with ASD.	Narrative review	Gastrointestinal abnormalities; behaviour.	Improves: behaviours and gastrointestinal symptoms (constipation and diarrhoea). Negative effects: Reduction in cortical bone thickness.	Children predisposed to gastrointestinal abnormalities could benefit from the GFCF diet. However, negative consequences for the use of the GFCF were evident.
Whiteley, 2015 [28]. Dallas	Nutritional management of (some) autism: a case for gluten- and casein-free diets.	N/A	Examine the effect of dietary intervention for improving ASD.	Literature review	The gut–brain link and autism and coeliac disease (CD).	Possible diet-related benefits on food metabolites, immune response, issues with gut barrier function and some contribution from the gut microbiota.	Calls for additional, well-designed studies are necessary to establish dietary effects.
Babinska et al., 2020 [29]. Slovakia	Gastrointestinal Symptoms and Feeding Problems and Their Associations with Dietary Interventions, Food Supplement Use, and Behavioural Characteristics in a Sample of Children and Adolescents with Autism Spectrum Disorders.	247 children and adolescents with ASD	Investigate the prevalence of GI symptoms, food selectivity, mealtime difficulties, and their associations with dietary interventions, food supplement use, and behavioural characteristics among individuals with ASD.	Case–control study	Gastrointestinal problems; mealtime behaviour; feeding issues.	Nutrient deficiencies.	No significant correlation between following a diet and the severity of GI symptoms or mealtime problems was found.
Elder et al., 2015 [30]. USA	A review of gluten- and casein-free diets for treatment of autism: 2005–2015.	N/A	Report the effectiveness and safety of the GFCF diet in the treatment of ASD.	Literature review	Cognitive function; behavioural issues.	Positive changes in cognitive function, interaction, and behaviour.	Clinicians should use caution when advising on the GFCF diet for individuals with ASD until rigorous research supporting its use is reported.
Van De Sande et al., 2014 [31]. Netherlands	Autism and nutrition: the role of the gut–brain axis.	N/A	Evaluate the current theories and hypotheses concerning the aetiology of autism, with a special focus on the gut–brain axis.	Literature review	Opioid excess theory; psychological and behavioural categories.	Improvement in psychological and behavioural categories. Improved communication, attention and concentration, episodes of aggressiveness, affection, motor skills, sleeping patterns, displaying of routines and rituals, anxiety, empathy and responses to learning.	Contradictory but promising results. Nutrition and environmental factors might contribute to the development of autism.
Piwowarczyk et al., 2018 [32]. Poland	Gluten- and casein-free diet and autism spectrum disorders in children: a systematic review.	6 RCTs 214 participants	Determine the effectiveness of a gluten-free and casein-free (GFCF) diet as a treatment for ASD in children.	Systematic review	Communication; social interaction.	Significant improvements in communication and social interaction. No adverse events.	Overall, there is little evidence that a GFCF diet is beneficial for the symptoms of ASD in children.
Lau et al., 2013 [33]. San Francisco	Markers of Celiac Disease and Gluten Sensitivity in Children with Autism.	140 children	Assess the immune reactivity to gluten in paediatric patients diagnosed with autism according to strict criteria and to evaluate the potential link between autism and celiac disease.	Case–control study	Celiac disease; gut–brain interface.	Gluten response was significantly greater in autistic children with gastrointestinal symptoms in comparison to those without them.	Inconclusive findings regarding the increased IgG antibody response to gliadin (gluten).
Hurwitz, 2013 [34]. Israel	The Gluten-Free, Casein-Free Diet and Autism: Limited Return on Family Investment.	N/A	Identify and evaluate well-controlled studies of the GFCF diet that have been implemented with children with ASD.	Literature review	Behavioural and developmental outcomes; opioid excess theory; GI difficulties.	Negative behavioural and developmental outcomes seen in ASD. No positive effects of the diet on behaviour or development (3 studies). Positive effects after 1 year but had research quality concerns (2 studies).	The GFCF diet does not significantly change functioning or behaviour for most children with ASD.
Alamri, 2020 [35]. Kingdom of Saudi Arabia	Efficacy of gluten- and casein-free diets on autism spectrum disorders in children.	9 RCT s 521 participants	Resolve uncertainty regarding the effect of GFCF diet on ASD.	Literature review	Communication; stereotyped movements; aggressiveness, hyperactivity, tantrums, and signs of ADHD.	Studies showed progress in certain traits: improvement in communication, stereotyped movements, aggressiveness, hyperactivity, tantrums, and signs of ADHD.	The data remains insufficient to support the use of GFCD to improve the symptoms of ASD in children.
González-Domenech et al., 2019 [36]. Spain	Influence of a Gluten-Free, Casein-Free Diet on Behavioural Disturbances in Children and Adolescents Diagnosed with Autism Spectrum Disorder: A 3-Month Follow-Up Pilot Study	28 patients	Investigate how a gluten-free, casein-free (GFCF) diet affects behaviour changes in ASD children and adolescents. Examine any potential correlation between ASD symptoms and the urinary levels of beta-casomorphin.	Cross-over clinical trial	ATEC scale: Communication; Cognitive awareness; Behaviour. ERC-III Scale: Communication; Motility; Attention, perception and intellectual functions. ABC Scale: Irritability and agitation; Lethargy; Social withdrawal; Hyperactivity.	A nonsignificant decrease in ATEC scores after the GFCF diet was found. No statistically significant differences in the ERC-III and ABS scales were found.	A three-month GFCF diet showed no significant changes in autism symptoms or urine beta-casomorphin (opioid peptide) levels.
El-Rashidy et al., 2017 [37]. Egypt	Ketogenic diet versus gluten free casein free diet in autistic children: a case–control study.	45 children	Compare the effect of the ketogenic diet as the modified Atkins diet (MAD) and GFCF diet in autistic children to eliminate core symptoms of autism.	Case–control study	Neurological examination; anthropometric measures. Childhood Autism Rating Scale (CARS) evaluating: Behaviour. Autism Treatment Evaluation Test (ATEC) scales: Communication; cognitive function.	Modified Atkins Diet (MAD) group: Significant improvement in speech, social and cognition parameters. Decrease in the severity of ASD symptoms. GFCF group: Improvement in total CARS and ATEC scores of speech and behaviour. Ketogenic diet: scored better results in cognition and sociability compared to the GFCF diet group.	MAD and GFCF diet regimens may safely improve autistic manifestations and could be recommended for children with ASD.
Marı’-Bauset et al., 2016 [38]. Spain	Nutritional Impact of a Gluten-Free Casein-Free Diet in Children with Autism Spectrum Disorder	105 children	Explore the effects of the GFCF diet on anthropometric and nutritional status and behavioural symptoms, in children with ASD.	Case–control study	Anthropometric values; nutritional status.	Lower anthropometric values and worsened nutritional status.	GFCF diet should be considered only after diagnosing any intolerance or allergy among autistic individuals.
Monteiro et al., 2020 [39]. Brazil	Autism spectrum disorder: A systematic review about nutritional interventions.	N/A	Analyse scientific evidence found in literature regarding nutritional interventions, including the GFCF diet, carried out in children and teenagers with ASD.	Systematic review	Communication: Stereotypic movements; gastrointestinal (GI) symptoms.	Improved communication, stereotyped movements, aggressiveness, signs of ADHD, and GI symptoms.	Progress in the symptoms associated with autism.
Keller et al., 2021 [40]. Denmark	The Effect of a Combined Gluten- and Casein-Free Diet on Children and Adolescents with Autism Spectrum Disorders: A Systematic Review and Meta-Analysis	N/A	Investigate the benefit and safety of a GFCF diet among children with a diagnosis of ASD	Systematic review and meta-analysis	Core autistic symptoms; gastrointestinal discomfort; behavioural difficulties.	Side effects: Gastrointestinal discomfort, weight loss. and loss of appetite, and sleep disturbance. Limitations in the current literature.	Reported side effects after following the GFCF diet. Well-designed, high-quality clinical trials of sufficient duration are recommended.
Cekici et al., 2019 [41]. Turkey	Current nutritional approaches in managing autism spectrum disorder: A review	N/A	Evaluating scientific evidence of existing medical nutrition therapies and their effects on alleviating ASD symptoms	Literature review	Opioid theory; ASD-related behaviours and hyperactivity; GI symptoms; attention and focus; speech and communication; sleeping issues.	Improved ASD symptoms and decreased hyperactivity behaviours. Decreased GI symptoms. Improvement in speech and communication skills. Decrease in sleep problems. Nutritional deficiencies and a possible decline in growth and development.	Contradictory results Further prospective controlled trials with large sample sizes are needed.
Akhter et al., 2022 [42]. Pakistan	A narrative review on manifestations of gluten-free casein-free diet in autism and autism spectrum disorders.	N/A	Assess the utility of the GFCF diet for the management of autism.	Narrative review	Autism symptoms; gastrointestinal disorders; emotional, behavioural, cognitive, and learning abilities.	Improvement in mental wellbeing, hyperactive behaviour and anxiousness; GI symptoms, cognitive function, learning abilities, developmental outcomes, and sleeping habits.	The GFCF diet, when combined with nutritional therapy, improves mental wellbeing in the affected children.
Adams et al., 2018 [43]. USA	Comprehensive Nutritional and Dietary Intervention for Autism Spectrum Disorder-A Randomised, Controlled 12-Month Trial	67 children and adults with ASD	Investigate comprehensive nutritional and dietary intervention to treat children and adults with ASD.	Randomised, controlled, single-blind 12-month study	Nutrition; social isolation; maintaining eye contact; learning skills; hyperactivity; panic attacks; stereotypic autistic patterns.	Improvement in nutritional status, communication, hyperactivity and panic attacks; improved congestive skills, autism stereotyped movements.	Effective at improving nutritional status, nonverbal IQ, autism symptoms, and other symptoms in most individuals with ASD.
Mendive Dubourdieu. et al., 2022 [44]. Uruguay	Dietary Intake, Nutritional Status and Sensory Profile in Children with Autism Spectrum Disorder and Typical Development	65 children	Analyse dietary intake, nutritional status, and sensory profile in children with and without ASD.	A descriptive, cross-sectional study	Nutrition status; autistic behaviours.	Two studies reported positive effects at the end of 1 year on the diet. Results could be impacted by placebo effects and high attrition rates.	Further long-term research is needed to explore their impact on health.
Pedersen et al., 2014 [45]. UK	Data mining the ScanBrit study of a gluten- and casein-free dietary intervention for children with autism spectrum disorders: Behavioural and psychometric measures of dietary response	72 children with ASD	Examination of a gluten- and casein-free diet as an intervention for children diagnosed with an autism spectrum	Case–control study	Hyperactivity behaviours.	Reduced symptoms of hyperactivity/impulsivity and inattention resulted from the GFCF diet.	Participants aged between 7 and 9 years seemed to derive the most benefit from dietary intervention.
Baspinar et al., 2020 [11]. Turkey	Gluten-Free Casein-Free Diet for Autism Spectrum Disorders: Can It Be Effective in Solving Behavioural and Gastrointestinal Problems?	N/A	Investigate the gastrointestinal and behavioural problems that are frequently observed in ASD, the possible action mechanisms of GFCF diets, and the efficacy of these elimination diets	Literature review	Opioid theory; gastrointestinal symptoms; concentration; autistic-related repetitive behaviours.	Support the opioid theory (reduced pain, sensitivity and altered social behaviour) Improved gastrointestinal symptoms, concentration and attention increased. Decreased stereotypical behaviours. Methodological limitations were observed.	Support the opioid theory, and is beneficial to reduce GI symptoms, as well as autistic-specific behaviours.
Hafid et al., 2018 [46]. Morocco	The Efficacy of the Gluten-Free Casein-Free Diet for Moroccan Autistic Children.	30 children with ASD	Verify the efficiency of gluten-free casein-free diet for children with autism spectrum disorder and to evaluate its impact on their nutritional profiles.	Experimental survey	Concentration; autistic symptoms severity.	Improved concentration and decreased autism severity. among 30% of participants.	Beneficial only to autistic children who present with both very high urinary peptide and gastrointestinal problems.
Lyra et al., 2017 [47]. Brazil	What do Cochrane systematic reviews say about interventions for autism spectrum disorders?	N/A	The manifestations of ASDs can have an important impact on learning and social functioning that may persist during adulthood. The aim was to summarise the evidence on interventions for ASDs	Review of systematic reviews	Autistic traits; communication.	Significant positive effect for autistic traits and the overall ability to communicate and interact (in two small RCTs (35 participants). No adverse reactions from GFCF diet.	Evidence suggests benefits for overall autistic traits and communication. However, the evidence was not robust enough to support the benefits of the GFCF diet.
Nurul Hakim et al., 2023 [48]. Indonesia	Effect of Gluten Free Casein Free Diet on Maladaptive Behaviour in Autistic Children: Meta Analysis.	N/A	Estimate the effect of a casein-free gluten-free diet on maladaptive behaviour in autistic children, through a meta-analysis of primary studies conducted by previous authors.	Meta-analysis	Maladaptive behaviour.	Improved behaviour.	Autistic children who received casein-free and gluten-free diet had lower maladaptive behaviour than those who were not given the diet.
Saad et al., 2024 [49]. Egypt	Gluten-free, casein-free diet for children with autism spectrum disorder: A case–controlled study.	36 children with ASD	Assess the effectiveness of the gluten-free, casein-free (GFCF) diet in a cohort of Egyptian children with ASD.	Case–control study	Autistic behaviours.	Improved autistic behaviours.	The implementation of the GFCF diet significant improvements in CARS scores after a 6-month and 1-year follow-up period.
Quan et al., 2022 [10]. China	Systematic review and meta-analysis of the benefits of a gluten-free diet and/or casein-free diet for children with autism spectrum disorder.	297 autistic children	To evaluate the efficacy and safety of a GFCF diet for children with ASD.	Systematic review and meta-analysis	Stereotypical autistic behaviours; cognition.	Improved stereotypical behaviours and cognition.	The GFCF diet can reduce stereotypical behaviours and improve the cognition of children with ASD. No statistically significant changes were observed in other symptomatic categories.
Obara et al., 2023 [50]. Kenya	A review of dietary and nutritional interventions available for management of autism spectrum disorders symptoms in children and adolescents—Kenya	1298 autistic children	To identify dietary and nutritional interventions available for the management of ASD symptoms in children and adolescents	Systematic review	Communication; stereotypical movement; aggressiveness; hyperactivity; ADHD; gastrointestinal disorders.	Improvement in communication, stereotypical movements, aggressive behaviour, language hyperactivity, tantrums, attention deficit hyperactivity disorder (ADHD), and gastrointestinal disorders.	GFCF diet is beneficial in improving communication, stereotypical movement, speech, ADHD, and gastrointestinal issues.

## Data Availability

All data/studies used in this review are listed in the reference list.
